# Field Testing the “Avoid the Needle” Intervention for Persons at Risk for Transitioning to Injecting Drug Use in Tallinn, Estonia and New York City, USA

**DOI:** 10.1007/s10461-023-04094-0

**Published:** 2023-05-30

**Authors:** Don C Des Jarlais, Courtney McKnight, Chenziheng Allen Weng, Jonathan Feelemyer, Susan Tross, Mait Raag, Greete Org, Ave Talu, Anneli Uuskula

**Affiliations:** 1 New York University School of Global Public Health, New York, NY USA; 2 Department of Psychiatry, Columbia University, New York, NY USA; 3 Department of Family Medicine and Public Health, University of Tartu, Tartu, Estonia; 4 OÜ ReCuro, Tallinn, Estonia

**Keywords:** HIV/AIDS, People who use drugs (PWUD), Persons who inject drugs (PWID), Transitions to injecting drug use, New York City, Tallinn Estonia

## Abstract

This study aimed to field tested the “Avoid the Needle” (AtN) intervention to reduce transitions from non-injecting to injecting drug use in two different epidemiological settings. Respondent driven sampling was used to recruit current non-injecting drug users (NIDUs) in Tallinn, Estonia in 2018-19 and in New York City (NYC) in 2019-20. Both persons who had never injected and persons who had previously injected but not in the last 6 months were eligible; a structured interview was administered, a blood sample collected, and the intervention administered by trained interventionists. We recruited 19 non-injectors from Tallinn and 140 from NYC. Participants in Tallinn were younger and had begun using drugs at earlier ages than participants in NYC. The primary drugs used in Tallinn were amphetamine, fentanyl, and opioid analgesics, while in NYC they were heroin, cocaine, speedball, and fentanyl. Six-month follow-up data were obtained from 95% of participants in Tallinn. The study was interrupted by COVID-19 lockdown in NYC, but follow-up data were obtained from 59% of participants. There were minimal transitions to injecting: 1/18 in Tallinn and 0/83 in NYC. There were significant declines in the frequencies of using readily injectable drugs (fentanyl, amphetamine, heroin, cocaine) from baseline to follow-up in both sites (Cochran-Armitage tests for trend, χ^2^ = 21.3, p < 0.001 for New York City; and χ^2^ = 3.9, p = 0.048 for Tallinn). Reducing transitions into injecting is a potentially very important method for reducing HIV transmission and other harms of drug use. Further investigation and implementation of AtN type interventions is warranted.

## Introduction

There are an estimated 11.2 million persons who inject illicit psychoactive drugs (PWID) throughout the world [1]. The United States, China and Russia are all estimated to have PWID populations of 1 million or more [2]. Injecting is almost certainly the most dangerous route of psychoactive drug administration. Compared to non-injecting routes of administration, injecting greatly increases the likelihood of HIV and hepatitis C (HCV) transmission [3, 4], is more likely to rapidly lead to drug dependence [5], and is more likely to lead to fatal drug overdose [6]. Injecting may be a particularly dangerous route of drug administration in settings where there are: (1) a scarcity of sterile injecting equipment and high prevalence of blood-borne viruses, (2) lack of large-scale evidence-based treatment programs for the drugs being injected, and (3) particularly potent drugs, such as fentanyl, that create a high likelihood of overdose.

Once injecting has become well established as a route of drug administration in a local area, it is extremely difficult to reverse. New injectors are continuously recruited into injecting drug use, typically from persons using drugs through non-injecting routes of administration. Recruitment is typically done by current injectors [7, 8].

Despite the great increases in individual and societal harms associated with transitions from non-injecting to injecting drug use, there has been very little development of public health interventions to prevent or reverse these transitions. “Scare/fear arousal” programs and incarceration of persons who use drugs have not been effective [9, 10]. Treatment programs for substance use disorder (SUD) clearly have considerable potential for reducing transitions to injecting but in many areas of the world, treatment programs for non-injecting users are limited [11], and the programs usually emphasize abstinence which may not be a goal of persons who are at risk for transitioning to injecting.

We report here on two community-based studies of the Avoid the Needle (AtN) intervention, which was developed to reduce the likelihood that persons currently using drugs through non-injecting routes of administration would transition to injecting. The intervention addresses both initial transitions to injecting, and return (relapse) to injecting among persons who had injected in the past and then changed to non-injecting routes of drug administration.

### Intervention

“Avoid the Needle” (AtN) was adapted from the “Heroin Sniffer” project [12, 13], first developed in the 1990s with the purpose of reducing the likelihood that persons currently using heroin intranasally would initiate injection drug use. The Heroin Sniffer project was tested in a randomized controlled trial with individuals reporting any injecting drug use during the 8-month follow-up as the primary outcome. Injecting in the experimental group was lower than in the control group (adjusted odds ratio (aOR): 0.51, 95% CI: 0.27, 0.99). The Heroin Sniffer Project was included in the HIV/AIDS Prevention Program Archive compendium of effective HIV prevention interventions [14].

As with the original Heroin Sniffer intervention, the AtN intervention is based in social cognitive theory [15, 16], cognitive behavioral skills building, and motivational interviewing [17, 18] to enhance the participant’s own motivations and actions to not inject drugs. It was administered by masters or bachelor’s level counselors. The primary adaptation from Heroin sniffer to AtN was changing from four small group sessions to two one-on-one sessions. A secondary adaptation was to include non-injecting use of a wider variety of non-injected drugs that could be injected, such as fentanyl, cocaine, opioid analgesics, amphetamine, and methamphetamine. A third adaptation was to change the eligibility requirements for “former injectors” to have at least 6 months of non-injecting drug use since their last injection rather than only 2 months. Topics covered in the sessions included reviewing the personal history of drug use, discussing reasons for not injecting, awareness of activities that promote injecting, particularly interactions with current injectors, and skill practice in refusing encouragement to inject and offers of assistance with first injections. Short “booster sessions” (approximately 20 min) were held with participants one month after the initial intervention session to review the contents of the initial session and to reinforce participants’ motivation to avoid injecting.

The AtN intervention is a strength-based intervention in that it focused on the participants’ own motivations and skills to avoid injecting and is a harm reduction intervention in that the desired outcome was maintaining a safer form of drug administration (non-injecting use) rather than achieving abstinence.

### Pilot Testing

Pilot testing of AtN was conducted in Tallinn, Estonia, and in New York City (NYC), USA. These sites were selected to represent considerable differences in the epidemiology of injecting drug use but with well-developed research capabilities. The pilot testing did show low rates of transitions from non-injecting to injecting drug use during follow-up—lower than those observed in the initial randomized clinical trial of the Heroin Sniffer project—in both sites [8, 19]. The pilot work also raised several implementation issues. First, it was difficult to recruit large numbers of non-injectors. The substance use treatment programs in the two sites generally were not interested in having their clients participating in a harm reduction intervention to avoid transitions to injecting drug use, rather they emphasized abstinence as their primary treatment goal. Criminal justice systems also contain many non-injecting users but are also oriented to providing services focused on abstinence. Many vocational educational institutions recognized that their students used drugs and were at risk for injecting drug use but did not want to publicly acknowledge this situation.

Second, research studies that require time and effort from persons who use drugs are generally ethically obligated to provide compensation to participants. Former injectors—persons with histories of injecting but who were not currently injecting—are eligible to participate in AtN, but there is no easy method for distinguishing persons who are currently injecting from those who were currently using and had previously injected but were not currently injecting.

The present study addressed these implementation issues by using community-based respondent driven sampling (RDS) [20, 21] with cross-recruiting among persons who did and did not inject. This eliminated the motivation to falsely deny current injecting and increased the likelihood for recruiting non-injectors who had social ties to injectors, which would place them at higher risk for transitioning to injecting.

## Methods

### Settings: Tallinn, Estonia (2018-19)

Estonia is a small Baltic country with a population of approximately 1.3 million. It was part of the Union of Soviet Socialist Republics from until 1990 when it became an independent country. Estonia experienced a very large epidemic of injecting drug use beginning in the 1990s and a very high seroprevalence epidemic of HIV (i.e., greater than 50% prevalence) among PWID since the 2000s [22]. Heroin was originally the dominant drug injected, but was replaced by fentanyl in 2004 [23]. The use of fentanyl generated the highest fatal drug overdose rates in Europe [24, 25]. The fentanyl supply was interrupted in 2017 [26] when a large illicit laboratory was shut down, but has since returned. Amphetamine is also frequently injected. New injectors have exhibited high-risk behavior and considerable HIV prevalence [27]. Community Needle and Syringe Programs (NSP), methadone maintenance treatment, and naloxone distribution programs were operating in Tallinn at the time of the study execution. The proportion of PWID receiving ART in Tallinn has increased substantially over the years, reaching over 70% among HIV-infected PWID [22].

### Settings: New York City (2019–2020)

NYC experienced the world’s largest local HIV epidemic among PWID, with prevalence reaching 50–60% in the early 1980s [28]. By the early 2010s, “combined treatment and care” (syringe service programs, medicated assisted treatment including methadone and buprenorphine, and antiretroviral “treatment as prevention” for HIV seropositive PWID) had reduced HIV prevalence to less than 10% and incidence to less than 1/100 person-years [28].

NYC also experienced the US “opioid epidemic,” and in the late 2010s was in the fentanyl phase [29], in which illicitly manufactured fentanyl had entered the drug distribution system. Fentanyl was often mixed with both heroin and cocaine in NYC [30], and users often did not know that fentanyl was being mixed with, or even substituted for, the illicit drugs they were purchasing. Fatal drug overdose greatly increased during the fentanyl phase [31].

### Research Design

The study was designed as two parallel single arm trials of the AtN intervention to assess whether it could be scaled up through community-based respondent driven sampling (RDS). Efficient recruiting of large numbers of non-injecting drug users would be essential for scale-up implementation of the intervention. The primary endpoint for both trials was any illicit drug injection during the 6-month post-intervention follow-up. Results were compared to illicit drug injection in the experimental and control arms of the original Heroin Sniffer study.

### Subject Recruitment

Respondent driven sampling **(**RDS) [20, 21] was used to recruit participants. Recruitment began with purposive selection of “seeds” known to the field teams to represent PWUD diverse by age, gender, ethnicity, main type of drug used, HIV status, length of drug using career, and main mode of drug administration. After study participation, subjects were provided coupons for recruiting three (in Tallinn) or up to six (in NYC) drug using peers. Coupons were uniquely coded to link participants to their survey responses and to biological specimens, and for monitoring recruitment lineages. Recruited peers had to come to the study site, be found eligible, and complete the study procedures for the recruiter to receive the secondary incentive. In both sites, persons currently injecting drugs (PWID) and persons using drugs without injecting (non-injecting drug users, NIDUs) were eligible to be recruited by and to recruit either PWID or NIDUs. We did not utilize RDS weighting in order to facilitate direct comparison of results across the two sites and comparison with the original Heroin Sniffer study.

### Tallinn

From December 2018 to April 2019 persons currently use illicit drugs (other than cannabis) were recruited through RDS [20, 21] in Tallinn. The NSP of NGO Convictus (fixed site) was the study site, given that: (1) it has established contacts and working experience with PWUD; (2) it provides HIV prevention services and is trusted by the PWUD community; (3) the site leader and staff have a track record of conducting research, including participation in international research teams, and have undergone extensive training in the conduct of scientific research.

### New York City

From December 2019 to March 2020, persons who were currently using illicit drugs other than marijuana were recruited through RDS in NYC. The study was located in a university building in southern Manhattan.

### Eligibility Criteria

Potential participants were eligible for the study if they: lived in the region (Tallinn or Harju County in Tallinn, and in the metropolitan area in NYC), were at least 18 years of age, spoke Estonian or Russian in Tallinn, or English in NYC, reported using illicit drugs other than marijuana, agreed to provide a blood sample for HIV testing, and were able and willing to provide informed consent.

### Study Procedures

Written informed consent was obtained followed by an interviewer-administered electronic structured survey. The survey included demographic characteristics, drug use history, current (past 6 months) drug use, HIV risk behavior and various health topics including drug overdose and psychological distress measured by the Kessler 6 scale [32]. We also included questions on whether the participants experienced “injection promoting behaviors”. These are behaviors that are likely increase the probability that a person using drugs might transition to injecting, and included (1) observing others inject, (2) hearing others talk positively about injecting, and (3) being helped with injecting.

HIV and HCV counseling were conducted, and a blood sample was collected for HIV and HCV testing, commercially available tests (ADVIA Centaur CHIV Ag/Ab Combo [SIEMENS]) in Tallinn. HIV testing was conducted at the New York City Department of Health laboratory using a commercial, enzyme-linked, immunosorbent assays (EIA) test with Western blot confirmation (BioRad Genetic Systems HIV-1–2 + 0 EIA and HIV-1 Western Blot, BioRad Laboratories, Hercules, CA). HCV testing was also conducted at the New York City Department of Health laboratory using the Abbott HCV enzyme immunoassay (EIA) 2.0 test.in NYC.

The first AtN intervention session was conducted immediately after the baseline data collection. Appointments were then made for a second intervention booster session at one month and a follow-up interview at 6 months. Follow-up information, including address, phone number and the name and contact information for another person who was likely to know how to contact the participant was collected.

### COVID-19 Changes in Study Procedures in New York

The AtN field trial coincided with the first year of the COVID-19 pandemic behavioral restrictions that occurred in NYC beginning in March 2020. In March 2020 COVID-19 pandemic restrictions required ceasing recruitment of new participants and ending in-person interviewing for current participants. This involved contacting participants who had appointments for in-person follow-up visits and informing them that the study had been halted with uncertainty about when it could be restarted. We then adapted the study to telephone interviewing, which involved obtaining new consents by telephone and distributing remote payment electronic debit cards by mail. The development, IRB approval, and implementation of these procedures required several months. To maintain rapport with subjects on the telephone, we shortened the original AtN follow-up survey, focusing transitions to injecting and efforts to avoid COVID-19 infection.

### Honoraria

#### Tallinn

Participants received a 20-euro grocery store voucher for their time and effort in the baseline interview, a 5-euro voucher for each new participant they recruited, and a 20-euro voucher for the follow-up interview.

#### New York City

Participants were compensated $30 for their time and effort in the baseline interview, $30 for time and effort in the intervention, and $5 for each new subject that they recruited for the study (all prior to the COVID-19 lockdown). During the COVID-19 lockdown, participants were compensated $20 per telephone interview through electronic transfers to the debit cards that had been mailed to them.

#### Ethical Review

The Tallinn component of the study was approved by the Research Ethics Committee (Institutional Review Board) of the University of Tartu. The NYC component of the study was approved by the Institution Review Board (IRB) of New York University (NYU) Langone Medical Center. The COVID-19 related modifications in follow-up procedures were also approved by the NYU Langone Medical Center IRB.

## Results

### Participants Recruited

Between Sept. 2019 and March 2020, 140 NIDUs from the five NYC boroughs were interviewed and received the AtN intervention. Between June 2020 and December 2020, we were able to recontact and interview 83 NIDUs for the follow-up interview. Many of the NYC participants appeared to have changed addresses and/or not been able to maintain their cell phones. The Estonia sample consisted of 19 NIDUs who received their baseline interviews and the AtN intervention between December 2018 to April 2019. The follow-up interviews (N = 18) were conducted between June 2019 and January 2020, with only one subject lost to follow-up.

### Demographics and Drug Use Behaviors

Participant demographics and drug use behaviors are presented in Table 1. There are clear differences in demographics and drug use. Participants in NYC were older and had been using drugs for longer periods of time. The racial/ethnic composition of the samples reflected the two different settings, although in both sites a majority of the participants belonged to minority groups within the cities: Blacks and Hispanics in NYC and ethnic Russians in Tallinn. A much larger percentage reported government benefits as their main source of income in NYC. The NYC sample and Tallinn sample had similar percentages of participants (36% and 37%, respectively) reporting secure housing, but the NYC participants were more likely to dwell in publicly supported housing including shelters and welfare residences, whereas the Tallinn participants featured a high proportion of being housed with daily room rentals. A much larger percentage were HIV seropositive in Tallinn (32%) than in NYC (7%).

**Table 1:** Baseline demographics and drug use related behaviors among non-injection drug use participants in New York City, USA and Tallinn, Estonia

Variable	New York City N = 140	Tallinn, Estonia N = 19
Demographics		
Biological sex (%)		
Males	111 (79%)	14 (74%)
Female	29 (21%)	5 (26%)
Mean Age (SD, range)	55 (9, 24–77)	32 (9, 19–50)
Race or ethnicity (%)		
Non-Hispanic Black	97 (69%)	
Non-Hispanic White	10 (7%)	
Hispanic	24 (17%)	
Mixed/Other race	9 (6%)	
Russian		16 (84%)
Estonian		3 (16%)
Education (%)		
No	43 (31%)	7 (37%)
Have High school diploma or GED	97 (69%)	12 (63%)
Main source of income in last 6 months (%)		
Regular job/employed with a regular salary	12 (9%)	6 (32%)
Spouse, partner, friend, or relative’s income/Other income	6 (4%)	0 (0%)
Temporary work, including legal odd jobs, off-books, etc.	16 (11%)	6 (32%)
Legal self-employment, including bottle deposits	5 (4%)	5 (26%)
Savings	0 (0%)	2 (11%)
Welfare, disability, Supplemental Security Income (SS)	101 (72%)	0 (0%)
Housing status in last 6 months (%)		
Their own (or partner’s) house, flat, apartment	51 (36%)	7 (37%)
Room rented on daily basis (hotel, rooming house)	13 (9%)	5 (26%)
Someone else’s (parents, relatives, friends) residence	36 (26%)	5 (26%)
Public housing (shelter, welfare residence)/Other housing	35 (25%)	1 (5%)
No fixed address (in the street, park)	3 (2%)	0 (0%)
Incarcerated (prison, house arrest)	2 (1%)	1 (5%)
Drug use history		
Mean age of first drug use (SD, range)	19 (6, 10–49)	17 (3, 9–23)
Injecting history (%)		
Never injected	83 (59%)	9 (47%)
Former injectors	57 (41%)	10 (53%)
Last time injected drugs among former injectors (%)		
More than 6 months ago to 12 months ago	3 (2%)	6 (60%)
More than 1 year ago to 5 years ago	15 (11%)	3 (30%)
More than 5 years ago to 10 years ago	8 (6%)	1 (10%)
More than 10 years ago	31 (22%)	0 (%)
Median number of injectors in the social networks (IQR)	4 (9)	5 (8.5)
Main drug^1^ (%)		
Heroin	76 (54%)	0 (0%)
Cocaine/crack	42 (30%)	6 (32%)
Speedball	0 (0%)	0 (0%)
Fentanyl	0 (0%)	1 (5%)
Amphetamine	0 (0%)	8 (42%)
Opiate analgesics	4 (3%)	1 (5%)
Other drugs	18 (13%)	3 (16%)
Non-injecting frequency of using main drugs (%)		
Once a week or less	30 (21%)	12 (63%)
Several times per week	55 (39%)	2 (11%)
Daily or more frequently	55 (39%)	5 (26%)
Current non-injecting use of readily injectable drugs^2^ (%)		
Not using	4 (3%)	0 (0%)
Currently using	136 (97%)	19 (100%)
Sexual behaviors		
Sexual orientation (%)		
Heterosexual or straight	128 (91%)	19 (100%)
Homosexual or bisexual	12 (9%)	0 (0%)
Number of people they had intercourse in last 6 months (%)		
0	44 (31%)	3 (16%)
1	69 (49%)	10 (53%)
> 1	27 (19%)	6 (32%)
Condom use frequency in last month among the sexually active (%)		
Not always	58 (60%)	14 (88%)
Always	38 (40%)	2 (12%)
Mental health		
Kessler Psychological distress (%)		
Moderate	110 (79%)	10 (53%)
Serious	30 (21%)	9 (47%)
Substance use disorder (%)		
Moderate	39 (28%)	7 (37%)
Severe	101 (72%)	12 (63%)
HIV/HCV		
HIV test result^3^ (%)		
Negative	129 (95%)	13 (68%)
Positive	7 (5%)	6 (32%)
HCV test result^3^ (%)		
Negative	108 (80%)	11 (58%)
Positive	27 (20%)	8 (42%)

^1^ Main drug is defined as participants’ self-reported most important drug in the past six months. Other drugs that subjects used mainly include marijuana, sedatives/benzos, synthetic cannabinoids (“K2”), street methadone, and alcohol in New York City, and sedatives/benzos and ecstasy in Tallinn

^2^ Readily injectable drug is defined as using heroin, cocaine, speedball, crack, fentanyl, amphetamine, and opiate analgesics in the past 30 days

^3^ Data for HIV blood test results were not successfully collected due to collapsed veins for 4 subjects at New York City (N = 136). And data for HCV blood test results were not successfully collected due to collapsed veins for 5 subjects at New York City (N = 135)

There were differences and similarities in drug use across the two sites. More than half (54%) of NYC participants reported heroin being their main drug, compared to 0% in the Tallinn sample. In the Tallinn sample, 42% reported amphetamine as their main drug, versus 0% in the NYC sample. The Tallinn sample also reported less frequent use of their main drugs, with 63% of the participants reported using once a week or less compared to 21% in the NYC sample.

In both sites, approximately half of the respondents were former and half never injectors. The percentages using readily injectable drugs (heroin, cocaine, fentanyl, amphetamine, and opioid analgesics) were very high in both sites (97% in NYC and 100% in Tallinn) (Table 1).

### Retained Versus Lost to Follow-Up Comparisons for New York City Participants

As noted above, follow-up data were obtained from 18/19 (94%**)** participants from Tallinn. Because of the COVID-19 “lockdown” in March 2020, follow-up data were obtained from 83 to 140 in NYC. Table 2 presents a comparison of the participants followed versus participants lost to follow-up. Tests for equality of proportions and means show that the baseline and follow-up samples of the NYC sample remain proportionate for most of the baseline demographics, drug use behaviors, HIV/HCV infection, and other behavioral measures despite the reduced sample size. The subjects who received follow-up in NYC were more likely to be female (Chi-square test = 8.4, p = 0.004), and had greater housing stability (Fisher’s exact test: p < 0.001). Since we found a much higher follow-up retention rate among those who were stably housed (84%, as compared to 59% in the total sample), further analysis was conducted to compare the subsample of the 43 participants who had stable housing at baseline and were followed with the 8 respondents who had stable housing at baseline and were not followed. There was no significant difference between these two groups who were stably housed at baseline.

**Table 2:** Baseline demographics and drug use related behaviors among non-injection drug use participants by followed or lost to contact in New York City

Variable	Non-injectors at baseline N = 140			
	Lost to follow-up, N = 57	AtN Follow-up, N = 83	*Test Statistic*	p-value
Demographic				
Sex^1^			8.4	0.004
Male	52 (91%)	59 (71%)		
Female	5 (9%)	24 (29%)		
Mean age (SD)^2^	54 (9)	56 (9)	1.4	0.17
Race or ethnicity^3^			-	0.29
Non-Hispanic Black	35 (61%)	62 (75%)		
Non-Hispanic White	4 (7%)	6 (7%)		
Hispanic	14 (25%)	10 (12%)		
Mixed/Other	4 (7%)	5 (6%)		
Education^1^			0.9	0.35
No	20 (35%)	23 (28%)		
Have High school diploma or GED	37 (65%)	60 (72%)		
Main source of income in last 6 months^3^			-	0.58
Regular job/employed with a regular salary	4 (7%)	8 (10%)		
Spouse, partner, friend, or relative’s income/Other income	1 (2%)	5 (6%)		
Temporary work, including legal odd jobs, off-books, etc.	8 (14%)	8 (10%)		
Legal self-employment, including bottle deposits	3 (5%)	2 (2%)		
Savings	0 (0%)	0 (0%)		
Welfare, disability, Supplemental Security Income (SS)	41 (72%)	60 (72%)		
Housing status in last 6 months^3^			-	< 0.001
Their own (or partner’s) house, flat, apartment	8 (14%)	43 (52%)		
Room rented on daily basis (hotel, rooming house)	4 (7%)	9 (11%)		
Someone else’s (parents, relatives, friends) residence	19 (33%)	17 (20%)		
Public housing (shelter, welfare residence)/Other housing	22 (39%)	13 (16%)		
No fixed address (in the street, park)	3 (5%)	0 (0%)		
Incarcerated (prison, house arrest)	1 (2%)	1 (1%)		
Drug use status				
Injecting Status^1^			0.1	0.78
Never injected	33 (58%)	50 (60%)		
Former injectors	24 (42%)	33 (40%)		
Last time injected drugs (Former injectors only)^4^			3.8	0.052
More than 6 months ago to 12 months ago	3 (5%)	0 (0%)		
More than 1 year ago to 5 years ago	7 (12%)	8 (10%)		
More than 5 years ago to 10 years ago	4 (7%)	4 (5%)		
More than 10 years ago	10 (18%)	21 (25%)		
Current non-injecting use of readily injectable drugs^3^			-	> 0.99
No	2 (4%)	2 (2%)		
Yes	55 (96%)	81 (98%)		
Non-injecting drug use frequency^4^			0.002	0.97
Once a week or less	15 (26%)	15 (18%)		
Several times per week	17 (30%)	38 (46%)		
Daily or more frequently	25 (44%)	30 (36%)		
Sexual behaviors				
Sexual orientation^3^			-	0.62
Heterosexual or straight	54 (95%)	74 (89%)		
Homosexual or bisexual	3 (5%)	9 (11%)		
Number of people they had intercourse in last 6 months^1^			1.0	0.62
0	16 (28%)	28 (34%)		
1	28 (49%)	41 (49%)		
> 1	13 (23%)	14 (17%)		
Condom use frequency among sexually active in last month^1^			0.3	0.60
Not always	26 (63%)	32 (58%)		
Always	15 (37%)	23 (42%)		
Mental health				
Psychological distress (Kessler) at baseline^1^			0.6	0.45
Moderate/Minor	43 (75%)	67 (81%)		
Serious	14 (25%)	16 (19%)		
Substance use disorder^1^			0.5	0.47
Mild/Moderate	14 (25%)	25 (30%)		
Severe	43 (75%)	58 (70%)		
HIV/HCV				
HIV test result^3,5^			-	0.24
Negative	54 (98%)	75 (93%)		
Positive	1 (2%)	6 (7%)		
HCV test result^3,5^			-	0.16
Negative	40 (74%)	68 (84%)		
Positive	14 (26%)	13 (16%)		

SD: Standard deviation. AtN: “Avoid the Needle” intervention. Readily injectable drug is defined as using heroin, cocaine, speedball, crack, fentanyl, amphetamine, and opiate analgesics in the past 30 days. Pearson’s Chi-squared test and Fisher’s exact test for comparing proportions,

^1^ Chi-squared test

^2^ Wilcoxon rank-sum test (Note: Z score was reported for test statistic)

^3^ Fisher’s exact test (Note: Test statistics are not applicable)

^4^Cochran-Armitage trend test

^5^Data for HIV blood test results were not successfully collected due to collapsed vein for 4 subjects (N = 136); data for HCV blood test results were not successfully collected due to collapsed vein for 5 subjects (N = 135)

### Exposure to Injection Promoting Behaviors and Injecting Status During Follow-up

Table 3 presents information on exposure to “injection promoting behaviors” and transitions to injecting among respondents during the 6 months prior to baseline and during the 6-month follow-up period in Tallinn and the 6-to-9-month follow-up period in NYC. Large percentages of respondents reported exposure to injection promoting behaviors in the 6 months prior to the baseline interview—63% in NYC and 100% in Tallinn. This fell to 28% exposed to injection promoting behaviors during follow-up in Tallinn. (Questions on exposure to injecting promoting behaviors during follow were not asked in NYC due to the need to reduce the length of the interview; see Methods)

**Table 3:** Baseline and follow-up exposures to injection-promoting behaviors among NIDU who were followed up in New York City, USA and in Tallinn, Estonia

Variable	New York City^1^ N = 83			Tallinn, Estonia N = 18			
	Baseline	Follow-up		Baseline	Follow-up	Test Statistic*	p
**Exposed to any promoting behaviors last 6 months** ^**2**^	52 (63%)	-		18 (100%)	5 (28%)	10.1	0.001
People talk positively about injection	32 (39%)	-		7 (39%)	4 (22%)	0.4	0.51
People injected in front of them	45 (54%)	-		11 (61%)	5 (28%)	2.5	0.11
People offered to help injection	14 (17%)	-		5 (28%)	3 (17%)	0.2	0.62
**Asked injectors about injection last 6 months** ^**2**^	12 (14%)	-		3 (17%)	0 (0%)	1.3	0.25
**Transitioned to injecting** ^**3**^	-	0 (0%)		-	1 (6%)		0.18

^*^Test statistics for “exposed to any promoting behaviors in last six months” and “asked injectors about injection in last six months” are for Tallinn only; the Test for “transitioned to injecting” is compare NYC to Tallinn

^1^ Follow-up questions on the exposures to injection-promoting behaviors were not collected in the New York City sample due to the interruption of the COVID-19 pandemic

^2^McNemar’s test

^3^Fisher’s exact test

One of the 18 participants with follow-up data in Tallinn reported injecting drugs during the follow-up period. This participant was a former injector. None of the 83 subjects in NYC reported injecting during the follow-up period.

### Changes in Drug Use During Follow-up

**Fig. 1:**
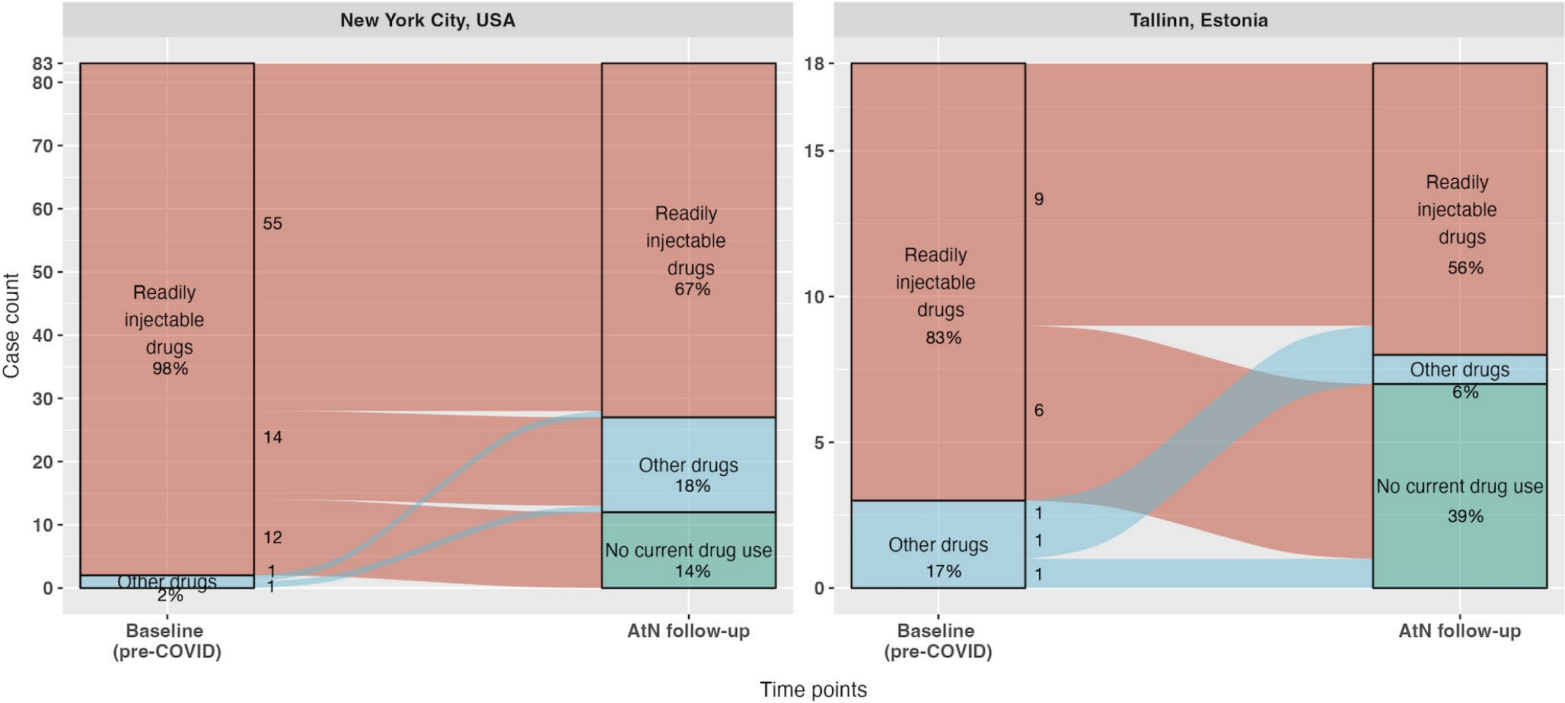
An alluvial plot of the distribution of the primary drug of subjects at baseline and at Avoid-the-Needle follow-up, multisite data from New York City, USA (N = 83) and from Tallinn, Estonia (N = 18). Readily injectable drugs were defined as using heroin, cocaine, speedball, crack, fentanyl, amphetamine, and opiate pills. Other drugs that subjects used include marijuana, alcohol, and sedatives/benzos at New York City, and sedatives/benzos and ecstasy at Tallinn. McNemar’s test for the proportion of using readily injectable drugs: χ^2^ = 21.3, p < 0.001 for New York City; and χ^2^ = 2.3, p = 0.13 for Tallinn

Figure 1 presents alluvial plots illustrating changes in the frequencies of respondents’ use of their main drugs from baseline to follow-up. Drugs were classified into “readily injectable” (heroin, cocaine, speedball, crack, fentanyl, amphetamine, and opioid analgesics) versus “other” to focus on possible transitions to injecting. Use of a readily injectable drug as primary drug was considered to increase the likelihood of a transition to injecting.

In NYC, 55 (66%) continued using a readily injectable drug as their main drug, 14 (17%) changed to a drug that was not readily injectable, 12 (14%) ceased drug use, and only 1 (1%**)** changed from other to readily injectable drugs (p < 0.001 by McNemar’s test). In Tallinn, 9 (50%) persons continued using readily injectable drugs, 6 (33%) participants changed from readily injectable drugs to ceasing drug use, and only 1 (6%) changed from other to readily injectable drugs (p = 0.13 by McNemar’s test).

Overall, the use of readily injectable drugs as one’s main drug fell in both sites, from 98 to 67% (p < 0.001 by McNemar’s test) in New York City and from 83 to 56% (p = 0.13 by McNemar’s test) in Tallinn.

**Fig. 2:**
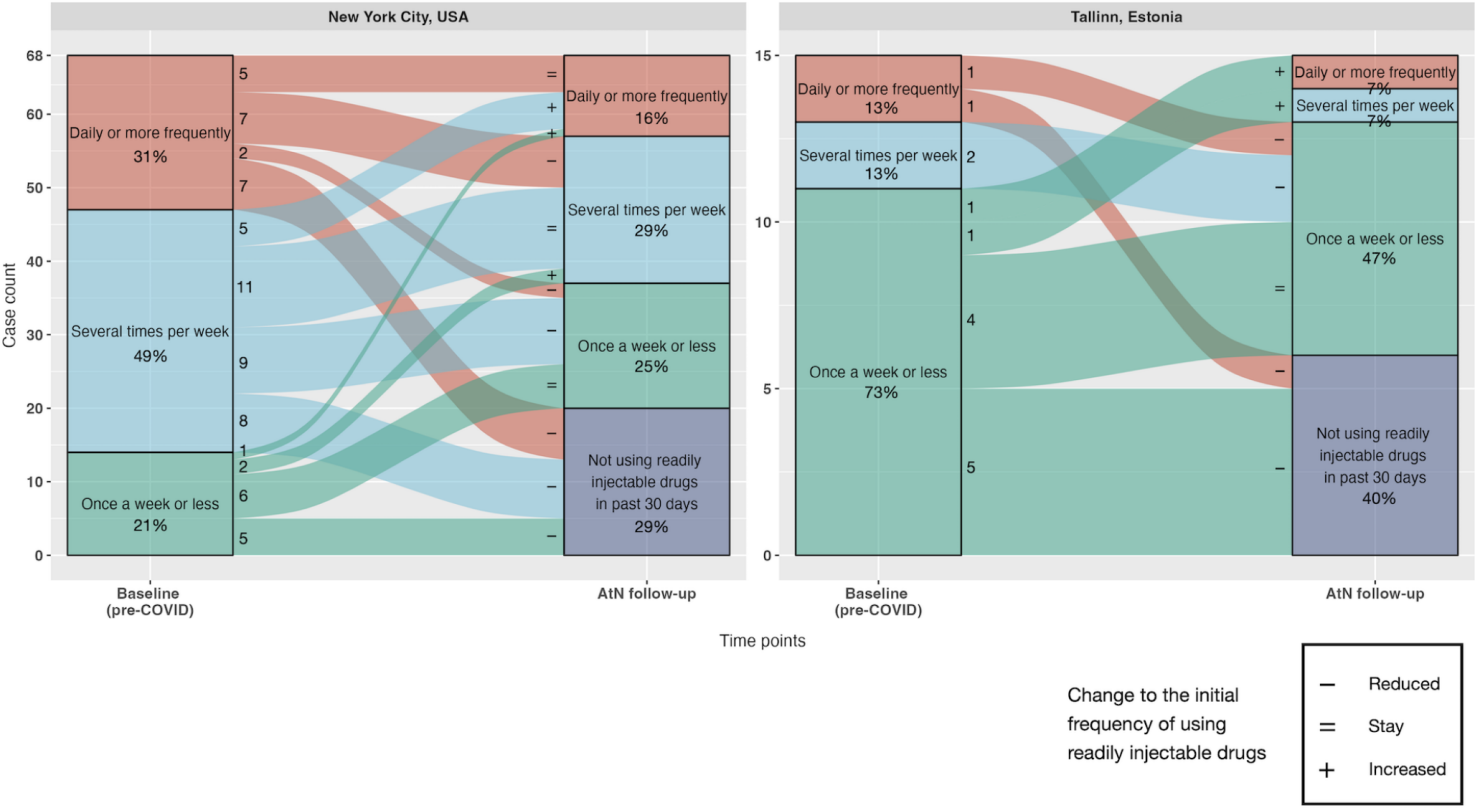
An alluvial plot of frequencies of using readily injectable drugs for subjects who were using readily injectable drugs as their main drug during Avoid-the-Needle follow-up, multisite data from New York City, USA (N = 68) and from Tallinn, Estonia (N = 15). Readily injectable drugs were defined as using heroin, cocaine, speedball, crack, fentanyl, amphetamine, and opiate pills. Cochran–Armitage test for the trend: χ^2^ = 21.3, p < 0.001 for New York City; and χ^2^ = 3.9, p = 0.048 for Tallinn

In addition to changes between readily injectable and other drugs, participants also reported changes in their frequency of use of readily injectable drugs. Figure 2 presents alluvial plots of the frequencies of use of readily injectable drugs for respondents who reported use of readily injectable drugs as their primary drug at baseline. There were 68 such respondents in NYC; 38 (56%) reported declines in frequency, including 20 (29%) who ceased using readily injectables, 22 (32%) reported using at the same frequency, and 8 (12%) who reported an increased frequency of use. In Tallinn, 15 respondents reported using readily injectables as their primary drug at baseline. Of these, 9 (60%) reported reduced frequency of use, including 6 (40%) who reported ceasing use of readily injectables, 4 (27%) reported using at the same frequency and 2 (13%) reported increased frequency of use. Overall, the frequency of using readily injectable drugs fell in both NYC (Cochran–Armitage test = 21.3, p < 0.001) and Tallinn (Cochran–Armitage test = 3.9, p = 0.048).

From baseline to follow-up, there was movement away from readily injectable drugs and toward less frequency use of readily injectables among respondents who were using readily injectables as their main drug at baseline.

## Discussion

### Implementation Issues

The use of RDS to simultaneously recruit both injectors and non-injectors addressed several implementation issues for working with non-injectors. It permitted efficient recruiting of a large number of non-injectors in NYC and a modest number in Tallinn. The non-injectors were at meaningful risk for transitioning to injecting in both sites; almost all were using drugs that could readily be injected with many using daily or more frequently, they had injectors in their social networks, a majority had serious SUDs, approximately half had injected in the past, and a majority had recently been exposed to injection promoting behaviors.

Additionally, simultaneously conducting different intervention studies with injectors and non-injectors reduced motivation to falsely report injecting/non-injecting status in order to participate in a study. These implementation objectives were achieved in both sites despite the many differences in the epidemiology of injecting and non-injecting drug use across the two sites.

### Primary Intervention Outcome

There were minimal transitions to injecting drug use among the successfully followed subjects at both sites: 1/18 (6%) in Tallinn and 0/83 (0%) in NYC. (The difference in transition percentage between the two sites is not significant, p = 0.18 by Fisher’s exact test). The transition percentages were lower than the 15% (6/40) observed in the follow-up period in the experimental arm of the original Heroin Sniffer study [12]. This difference may be due to the modifications of the intervention to a one-on-one intervention in which the respondent has the sole attention of the interventionist, addressing overdose risk in the intervention, and changes in the epidemiology of drug use over time in the sites.

### Reductions in the Use of Readily Injectable Drugs

As noted in the introduction, AtN is a harm reduction intervention intended to prevent transitions to a more harmful route of drug administration; it is not a substance use treatment intended to reduce drug use or achieve abstinence. Nevertheless, we did observe significant reductions in frequencies of the use of readily injectable drugs, including ceasing use of readily injectable drugs among 29% of participants in NYC and 40% of participants in Tallinn. These findings are consistent with the literature on motivational interviewing [33, 34] and brief interventions [35, 36]. From our clinical experience, the opportunity to reflect on one’s own drug use in a non-judgmental setting is an important aspect of the intervention.

### COVID-19 Pandemic in NYC

A few comments on the possible effects of the COVID-19 pandemic and behavioral restrictions in NYC are needed. The pandemic and restrictions disrupted drug supplies in NYC [37] and undoubtedly increased psychological stress for many persons who used drugs. Both of these factors could potentially increase transitions to injecting. Previous studies of drug shortages (“droughts”), however, have often shown that users decrease their drug use during these periods [38]. The pandemic restrictions (social distancing and stay at home guidance) also would have reduced interactions between non-injectors and injectors, which could have had a protective effect against transitions to injecting.

We obviously do not know the outcomes for the participants whom we were not able to contact during the COVID-19 pandemic but would note that we did have an 84% follow-up rate for the 51 NYC participants who were stably housed at baseline and none of these participants transitioned to injecting.

### Further Research

Long-term studies with random assignment control groups would be very helpful in further quantifying effect sizes and effect duration for the AtN intervention. Such studies should also investigate the potential mechanisms for reductions in the use of readily injectable drugs that occurred at both sites.

The results of the AtN interventions suggest that programs to encourage transitions from injecting to non-injecting drug use might also be successful.

### Limitations

Several limitations need to be mentioned. The lower follow-up rate due to the disruptions of the COVID-19 pandemic in NYC is of concern. However, an acceptable (84%) follow-up rate was achieved among participants who were stably housed at baseline in NYC. Also, the results were similar—minimal transitions to injecting—across all of the follow-up rates (95% in Tallinn, 84% among stably housed in NYC, and 59% among all NYC participants.

Data on drug use histories and drug use during follow-up was necessarily collected through self-report so that social desirability responding needs to be considered. The intervention focused on avoiding transition to injecting and not on reducing drug use per se, so that the reductions in self-reported drug use would not have been in response to demand characteristics of the intervention.

## Conclusions

Simultaneous RDS recruitment of both non-injectors and injectors, with cross-group recruiting led to relatively efficient recruiting of non-injectors with minimal incentives to falsely report injecting status. The non-injectors at both sites were at relatively high risk of transition to injecting, with approximately half having injected in the past, and a majority have been recently exposed to injecting promoting behaviors.

There were minimal transitions to injecting during the follow-up periods: 0/83 in NYC and 1/18 in Tallinn. There were also significant reductions in the use of “readily injectable drugs” (heroin, cocaine, speedball, fentanyl, amphetamine, and opioid analgesics) during follow-up.

Injecting is almost certainly the most harmful route of administration of illicit drugs and further investigation and implementation of AtN type interventions is warranted.

## Data Availability

All data in the analysis are available from the primary author.

## Code Availability

Code availability for the analysis is available from the primary author.

## References

[1] United Nations Office on Drugs and Crime. World Drug Report 2022. Vienna: 2022.

[2] UNODC. Number and prevalence of PWID and those living with HIV among this group 2017. Available from: https://dataunodc.un.org/drugs/pwid_hiv-2017.

[3] Mathias R (1991). Heroin snorters risk transition to injection drug use and infectious disease. NIDA Notes.

[4] Zibbell JE, Asher AK, Patel RC, Kupronis B, Iqbal K, Ward JW (2018). Increases in Acute Hepatitis C virus infection related to a growing opioid epidemic and Associated Injection Drug Use, United States, 2004 to 2014. Am J Public Health.

[5] Arria AM, Fuller C, Strathdee SA, Latkin C, Vlahov D (2002). Drug dependence among young recently initiated injection drug users. J Drug Issues.

[6] Davidson PJ, McLean RL, Kral AH, Gleghorn AA, Edlin BR, Moss AR (2003). Fatal heroin-related overdose in San Francisco, 1997–2000: a case for targeted intervention. J Urb Health.

[7] Khobzi N, Strike C, Cavalieri W, Bright R, Myers T, Calzavara L (2009). A qualitative study on the initiation into injection drug use: necessary and background processes. Addict Res Theory.

[8] Uusküla A, Barnes DM, Raag M, Talu A, Tross S, Des Jarlais DC (2018). Frequency and factors associated with providing injection initiation assistance in Tallinn, Estonia. Drug Alcohol Depend.

[9] Dolan K, Khoei EM, Brentari C, Stevens A. Prisons and Drugs: A global review of incarceration, drug use and drug services. Report 12. 2007.

[10] Springfield I, First P. Using Fear Messages and Scare Tactics in Substance Abuse Prevention Efforts.

[11] Des Jaralis DC, Casriel C, Friedman SR, Rosenblum A (1992). AIDS and the transition to illicit drug injection—results of a randomized trial prevention program. Br J Addict.

[12] Casriel C, Des Jarlais DC, Rodriguez R, Friedman SR, Stepherson B, Khuri E (1990). Working with heroin sniffers: clinical issues in preventing drug injection. J Subst Abuse Treat.

[13] Des Jarlais DC, Casriel C, Friedman SR, Rosenblum A (1992). AIDS and the transition to illicit drug injection: results of a randomized trial prevention program. Br J Addict.

[14] Card JJ, Benner T, Shields JP, Feinstein N (2001). The HIV/AIDS Prevention Program Archive (HAPPA): a collection of promising prevention programs in a box. AIDS Educ Prev.

[15] Bandura A (1986). Social foundations of thought and action: a social cognitive theory.

[16] Bandura A, Peterson J, DiClemente R (1993). Social Cognitive Theory and Exercise of Control over HIV infection. Preventing AIDS: theory and practice of behavioral interventions.

[17] Baker A, Dixon J, Miller WR, Rollnick S (1991). Motivational interviewing for HIV Risk reduction. Motivational interviewing: preparing people to change addictive behavior.

[18] Miller WR (1996). Motivational interviewing: Research, practice, and puzzles. Addict Behav.

[19] Des Jarlais DC, Uuskula A, Talu A, Barnes DM, Raag M, Arasteh K (2019). Implementing an updated “Break the Cycle” intervention to reduce initiating persons into injecting drug use in an eastern european and a US “opioid epidemic” setting. AIDS Behav.

[20] Heckathorn DD (2002). Respondent-driven sampling II: deriving valid population estimates from chain-referral samples of hidden populations. Soc Probl.

[21] Heckathorn DD (1997). Respondent-driven sampling: a new approach to the study of hidden populations. Soc Probl.

[22] Coombs R, Welles S, Hooper C, Reichelderfer P, D’Aquila R, Japour A (1996). Association of plasma human immunodeficiency virus type 1 RNA level with risk of clinical progression in patients with advanced infection. J Infect Dis.

[23] Platt L, Bobrova N, Rhodes T, Uusküla A, Parry JV, Rüütel K (2006). High HIV prevalence among injecting drug users in Estonia: implications for understanding the risk environment. AIDS.

[24] Jannetto PJ, Helander A, Garg U, Janis GC, Goldberger B, Ketha H (2019). The fentanyl epidemic and evolution of fentanyl analogs in the United States and the European Union. Clin Chem.

[25] Seyler T, Giraudon I, Noor A, Mounteney J, Griffiths P (2021). Is Europe facing an opioid epidemic: what does European monitoring data tell us?. Eur J Pain.

[26] Uusküla A, Talu A, Vorobjov S, Salekešin M, Rannap J, Lemsalu L (2020). The fentanyl epidemic in Estonia: factors in its evolution and opportunities for a comprehensive public health response, a scoping review. Int J Drug Policy.

[27] Des Jarlais DC, Friedman SR, Perlis T, Chapman TF, Sotheran JL, Paone D (1999). Risk behavior and HIV infection among new drug injectors in the era of AIDS in New York City. J J Acquir immune Defic Syndr Hum retrovirology.

[28] Des Jarlais DC, Kerr T, Carrieri P, Feelemyer J, Arasteh K (2016). HIV infection among persons who inject drugs: ending old epidemics and addressing new outbreaks. AIDS.

[29] Ciccarone D (2019). The triple wave epidemic: supply and demand drivers of the US opioid overdose crisis. Int J Drug Policy.

[30] McKnight C, Des Jarlais DC (2018). Being “hooked up” during a sharp increase in the availability of illicitly manufactured fentanyl: adaptations of drug using practices among people who use drugs (PWUD) in New York City. Int J Drug Policy.

[31] Colon-Berezin C, Nolan ML, Blachman-Forshay J, Paone D (2019). Overdose deaths involving fentanyl and fentanyl analogs—New York City, 2000–2017. Morb Mortal Wkly Rep.

[32] Andrews G, Slade T (2001). Interpreting scores on the Kessler psychological distress scale (K10). Aust N Z J Public Health.

[33] Miller WR, Yahne CE, Tonigan JS (2003). Motivational interviewing in drug abuse services: a randomized trial. J Consult Clinical Psychology.

[34] Carroll K, Ball S, Nich C, Martino S, Frankforter T, Farentinos C (2006). Motivational interviewing to improve treatment engagement and outcome in individuals seeking treatment for substance abuse: a multisite effectiveness study. Drug Alcohol Depend.

[35] Madras BK, Compton WM, Avula D, Stegbauer T, Stein JB, Clark HW (2009). Screening, brief interventions, referral to treatment (SBIRT) for illicit drug and alcohol use at multiple healthcare sites: comparison at intake and 6 months later. Drug Alcohol Depend.

[36] McQueen J, Howe T, Allan L, Mains D. Brief interventions for heavy alcohol users admitted to general hospital wards (Review). 2009. 10.1002/14651858.CD005191.pub2 19588369

[37] Bennett AS, Townsend T, Elliott L (2022). The COVID-19 pandemic and the health of people who use illicit opioids in New York City, the first 12 months. Int J Drug Policy.

[38] Degenhardt L, Conroy E, Day C, Gilmour S, Hall W (2005). The impact of a reduction in drug supply on demand for and compliance with treatment for drug dependence. Drug Alcohol Depend.

